# Insights Into Global Antimicrobial Resistance Dynamics Through the Sequencing of Enteric Bacteria From US International Travelers

**DOI:** 10.1093/infdis/jiaf469

**Published:** 2025-09-24

**Authors:** Sushmita Sridhar, Colin J Worby, Ryan A Bronson, Sarah E Turbett, Elizabeth Oliver, Terrance Shea, Sowmya R Rao, Vanessa Sanchez, Margaret V Becker, Lucyna Kogut Holliday, Damien Slater, Jason B Harris, Maroya Spalding Walters, Allison Taylor Walker, Mark C Knouse, Daniel T Leung, Paul Kelly, Edward T Ryan, Regina C LaRocque, Ashlee M Earl

**Affiliations:** Division of Infectious Diseases, Massachusetts General Hospital, Boston, Massachusetts, USA; Department of Medicine, Massachusetts General Hospital, Boston, Massachusetts, USA; Department of Medicine, Harvard Medical School, Boston, Massachusetts, USA; Infectious Disease and Microbiome Program, The Broad Institute of MIT and Harvard, Cambridge, Massachusetts, USA; Infectious Disease and Microbiome Program, The Broad Institute of MIT and Harvard, Cambridge, Massachusetts, USA; Division of Infectious Diseases, Massachusetts General Hospital, Boston, Massachusetts, USA; Department of Medicine, Massachusetts General Hospital, Boston, Massachusetts, USA; Department of Pathology, Massachusetts General Hospital, Boston, Massachusetts, USA; Division of Infectious Diseases, Massachusetts General Hospital, Boston, Massachusetts, USA; Infectious Disease and Microbiome Program, The Broad Institute of MIT and Harvard, Cambridge, Massachusetts, USA; Department of Global Health, Boston University School of Public Health, Boston, Massachusetts, USA; Division of Infectious Diseases, Massachusetts General Hospital, Boston, Massachusetts, USA; Department of Medicine, Massachusetts General Hospital, Boston, Massachusetts, USA; Division of Infectious Diseases, Massachusetts General Hospital, Boston, Massachusetts, USA; Division of Infectious Diseases, Massachusetts General Hospital, Boston, Massachusetts, USA; Division of Infectious Diseases, Massachusetts General Hospital, Boston, Massachusetts, USA; Division of Infectious Diseases, Massachusetts General Hospital, Boston, Massachusetts, USA; Department of Pediatrics, Harvard Medical School, Boston, Massachusetts, USA; Division of Healthcare Quality Promotion, National Center for Emerging and Zoonotic Infectious Disease, Centers for Disease Control and Prevention, Atlanta, Georgia, USA; Division of Global Migration and Quarantine, Centers for Disease Control and Prevention, Atlanta, Georgia, USA; Division of Infectious Diseases, Lehigh Valley Health Network, Allentown, Pennsylvania, USA; Division of Infectious Diseases, Division of Microbiology and Immunology, University of Utah, Salt Lake City, Utah, USA; Division of Infectious Diseases, Bronx Care Center, Bronx, New York, USA; Division of Infectious Diseases, Massachusetts General Hospital, Boston, Massachusetts, USA; Department of Medicine, Massachusetts General Hospital, Boston, Massachusetts, USA; Department of Medicine, Harvard Medical School, Boston, Massachusetts, USA; Travelers’ Advice and Immunization Center, Massachusetts General Hospital, Boston, Massachusetts, USA; Division of Infectious Diseases, Massachusetts General Hospital, Boston, Massachusetts, USA; Department of Medicine, Massachusetts General Hospital, Boston, Massachusetts, USA; Department of Medicine, Harvard Medical School, Boston, Massachusetts, USA; Travelers’ Advice and Immunization Center, Massachusetts General Hospital, Boston, Massachusetts, USA; Infectious Disease and Microbiome Program, The Broad Institute of MIT and Harvard, Cambridge, Massachusetts, USA

**Keywords:** antibiotic resistance, international travel, genomic surveillance, extended spectrum beta lactamase, *Escherichia coli*

## Abstract

**Background:**

Antimicrobial resistance (AMR) is an urgent threat to public health, but gaps in surveillance limit the detection of emergent novel threats and knowledge about the global distribution of AMR genes. International travelers frequently acquire AMR organisms (AMROs) and thus may provide a window into AMR dynamics in otherwise poorly monitored regions and environments.

**Methods:**

To assess the utility of travelers as global AMR sentinels, we collected pre- and post-travel stool samples from 608 travelers between 2017 and 2019, which were screened for the presence of extended-spectrum beta-lactamase producing Enterobacterales, carbapenem-resistant Enterobacterales, and *mcr*-mediated colistin-resistant Enterobacterales. A total of 307 distinct AMROs were sequenced and analyzed in order to determine genotypic patterns and their association with geography and traveler behavior.

**Results:**

Travel-associated AMROs were overwhelmingly *Escherichia coli*, which exhibited considerable phylogenetic diversity regardless of travel region. However, the prevalence of resistance genes varied by region, with *bla*_CTX-M-55_ and *bla*_CTX-M-27_ significantly more common in isolates associated with South America and South-Eastern Asia, respectively. Plasmid reconstruction revealed the genomic neighborhood of *bla*_CTX-M-55_ frequently matched a motif previously linked to animal populations. The ColV plasmid, a driver of avian pathogenic *E. coli*, was found to be elevated in frequency in isolates acquired by travelers reporting animal contact. We identified novel variants of the *mcr-1* gene in strains acquired from Western Africa.

**Conclusions:**

Traveler pathogen genomic surveillance can provide insight on global AMR dynamics and emerging clinical threats. Ongoing efforts to track travel-acquired organisms could complement existing global AMR surveillance frameworks.

## Teaser

International travelers frequently acquire AMR organisms; sequencing these pathogens upon return can provide insight into otherwise unobserved global AMR dynamics. This US traveler study highlights global microbial diversity, acquired plasmids associated with animal contact, and detection of emerging resistance variants.

Antimicrobial resistance (AMR) is a pressing global health concern, with recent estimates showing that approximately 5 million deaths in 2019 were associated with drug resistant infections [1]. Global differences in antibiotic administration and bacterial transmission rates have contributed to a highly heterogeneous AMR burden worldwide, with higher rates of AMR infections in many lower- and middle-income countries (LMICs), and rates are predicted to increase [1, 2]. Extended-spectrum beta-lactamase-producing Enterobacterales (ESBL-PE) are considered particularly problematic; over 60% of antimicrobials used are beta-lactams [3, 4]. The ability to detect the emergence and spread of novel AMR threats is hampered by limited surveillance in many parts of the world [5]; genomic surveillance, in particular, is predominantly conducted in wealthy countries and in healthcare settings.

International travel is a risk factor for acquisition of AMR organisms (AMROs) [6] and plays a role in the global spread of antibiotic resistance genes (ARGs) [7]; up to 60% of travelers acquire ESBL-PE [8]. By inadvertently sampling from microbial reservoirs, including food, water, and environmental sources in diverse global destinations, travelers may act as sentinels for the emergence and spread of novel AMR threats. Genomic surveillance of travelers could thus provide a proxy insight into global AMR dynamics.

Studies of travel-acquired AMROs have typically involved culturing organisms from stool on selective media, followed by targeted PCR-based assessment of genetic resistance factors [8–11]. Few studies have explored genomic and phylogenetic patterns of travel-acquired MDROs to assess global dissemination of novel ARGs and virulence factors [12, 13]. While some recent studies have sequenced isolates from travelers, these have been small in scale [14–16], focused on specific travel destinations [13, 17, 18], or been restricted to symptomatic individuals post-travel [19–22].

Previously, in an untargeted metagenomic analysis of stool samples from U.S. international travelers, we identified widespread acquisition of *Escherichia coli* strains and ARGs across a range of destinations [23]. However, due to the complexity and limited resolution of metagenomic sequence data, we were unable to identify specific ARGs with nucleotide-level accuracy, or to link genes with their plasmid or chromosomal hosts; such data are essential to tracking ARG spread via clonal dissemination or horizontal gene transfer. Here, we sequenced and analyzed 307 AMRO isolates representing ESBL-PE, carbapenem-resistant Enterobacterales (CRE), and *mcr*-mediated colistin-resistant Enterobacterales (mcr-E), collected before and after international travel from our previously described cohort of US travelers [24]. We sought to assess the utility of travelers as “global sentinels”; specifically, determining whether regional AMR trends and emerging threats could be identified from whole genome sequencing of travel-acquired bacteria. Our findings revealed geographic patterns of ARG prevalence, associations between traveler behavior and presence of virulence factors, and highlighted the role of plasmids in disseminating risk-associated genes both locally and globally. Our study demonstrates that traveler genomic surveillance could complement existing efforts to monitor the global prevalence and spread of AMR.

## METHODS

### Study Design and Sample Collection

We recruited 608 participants at 5 US travel clinics affiliated with Global TravEpiNet [25] between 2017 and 2019, applying no exclusion criteria, as previously described [24]. Participants self-collected pre- and post-travel stool samples, which were immediately stored in Cary Blair medium and mailed to the Massachusetts General Hospital clinical microbiology laboratory screened for the presence of ESBL-PE, mcr-E and CRE (Supplementary Text). Resistant colonies were picked; multiple colonies were picked if there were distinct morphotypes. Recovered organisms underwent antimicrobial susceptibility testing (AST) against a panel of 18 antibiotics using the Vitek2 AST automated system (bioMérieux, Durham, NC). Healthcare providers used structured questionnaires to collect information on demographics, health, travel itineraries and activities, medications, and symptoms.

### Whole Genome Sequencing and Data Analysis

DNA was extracted and sequenced for 394 AMROs from 203 participants, including from 186/217 (86%) of travelers with travel-acquired organisms. Long read sequence data were additionally generated for a subset of isolates (294/394; 64%). See Supplementary Text for full details on DNA extraction, sequencing, filtering, and analysis.

While colonies were selected based on differential morphology, some sequenced isolates from the same sample were nearly identical. To avoid overrepresentation of highly similar strains from the same traveler, we filtered out isolates <50 SNPs from another isolate from the same host. Post-filtering, same-host pairwise distances ranged from 500 to 12 000 SNPs, and most pairs exhibited distinct AST profiles. A total of 86 isolates were removed on the basis of this filtering, leaving 307 isolates for analysis. Genomes were assembled and profiled for ARGs, plasmid content, as well as presence of virulence and stress factors (Supplementary Text).

### Statistical Analysis

We aggregated travel destinations into geographic regions according to the United Nations Statistics Division [26], grouping regions with fewer than 10 travelers together into an ‘Other’ category. All statistical tests were performed at the isolate-level rather than the traveler-level, since we were primarily interested in the characteristics of the microbial populations associated with geographic regions and environmental niches. We used generalized estimating equations to test associations between binary features (eg, gene presence, plasmid presence, antibiotic non-susceptibility) and both geographic region and traveler activities [27] (Supplementary Text). Models were fit for all gene-region or gene-activity pairs. The Benjamini–Hochberg procedure was performed on each set of results to control for global false discovery; resistance, virulence, and stress genes were considered separately.

## RESULTS

### Travel-Acquired AMROs are Phylogenetically Distinct From Pre-travel Strains

**Figure 1.**
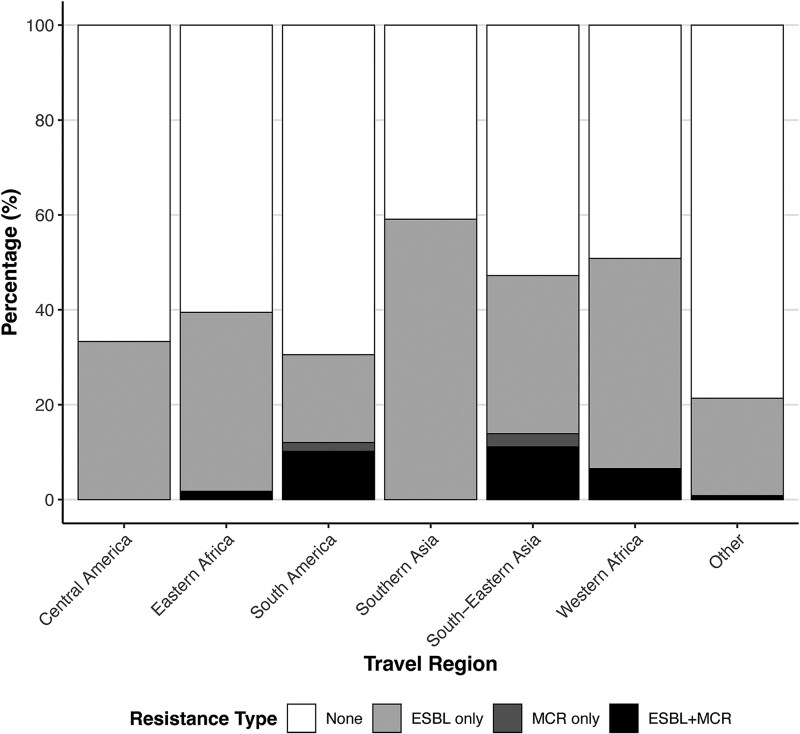
Global acquisition rates of AMRO organisms. For the 6 most common travel regions, stacked bars represent the proportions of travelers who returned carrying ESBL-PE and/or mcr-E. Regions are defined by the United Nations Statistics Division (*Methods*), and acquisition rates are calculated for the cohort of 568 travelers who were MDRO-negative pre-travel.

**Figure 2.**
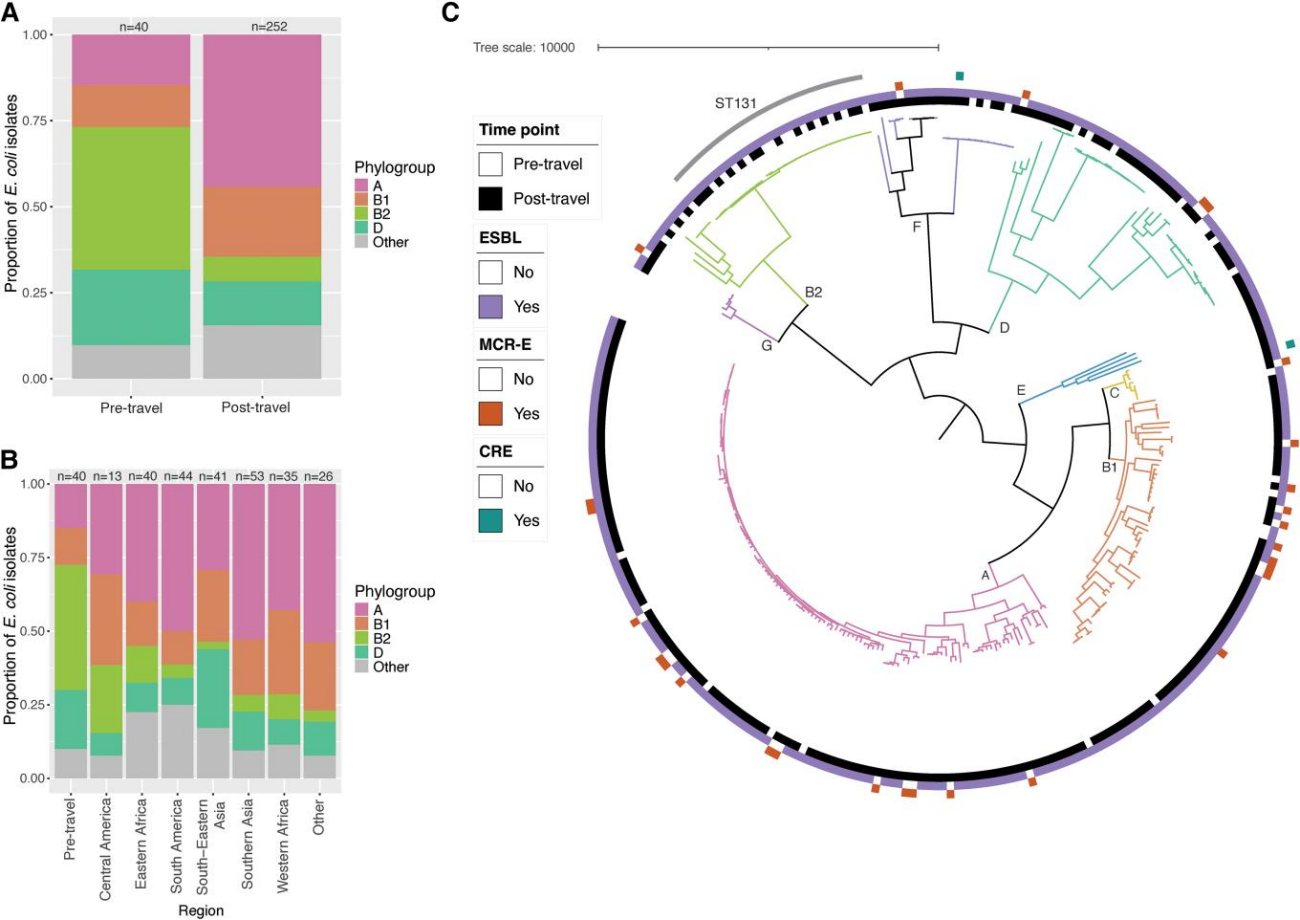
Phylogenetic distribution of acquired AMR *Escherichia coli*. The phylogroup designation for all 292 *E. coli* isolates is compared (*A*) between pre-travel and post-travel, and (*B*) between each of the most common travel regions. *C*, The midpoint-rooted phylogenetic tree is shown for all 292 *E. coli* isolates. The phylogeny was visualized in iTOL [28]; branches are colored and labeled by phylogroup.

As described in our previous study [24], 6.6% (40/608) of travelers were colonized with an AMRO before travel, and 38% (217/568) of those not colonized prior to travel returned with at least one AMRO, most often an ESBL-PE (98%, 212/217). There was considerable regional variability in AMRO acquisition rates (Figure 1). Of the 307 distinct AMRO genomes analyzed here (*Methods*), the majority (292/307, 95%) were *E. coli*, and most were ESBL-PE (285/307; 92.8%) (Table 1, Supplementary Table 1). Based on phylogroup, travel-acquired isolates were distinct from isolates carried pre-travel (Chi-squared test; χ^2^ = 42.95, *P* = 1.1×10^−8^); in particular, phylogroup B2 represented 43% (17/40) of pre-travel isolates, but only 7% (18/252) of travel-acquired isolates (Figure 2). Sequence type (ST) 131 (phylogroup B2) was overrepresented in pre-travel strains (odds ratio [OR] = 5.86, 95% CI: 2.52, 13.85; *P* = 4.2×10^−5^). Phylogroups A and B1 predominated post-travel (162/252; 64%); however, there were no significant differences in phylogroup distribution between travel regions among the post-travel isolates (Figure 2*B*). Travel associated isolates exhibited considerable ST diversity; of the 3 STs with more than 10 isolates, none were significantly overrepresented in any travel region.

**Table 1.** Summary of Traveler Isolates

	Count	%
**AMRO (n = 307)**	…	…
ESBL-PE	285	92.8%
MCR-E	28	9.1%
CRE	2	0.7%
**Species (n = 307)**	…	…
*E. coli*	292	95.1%
*K. pneumoniae*	10	3.3%
Other	5	1.6%
**Region (n = 307)**	…	…
Pre-travel	45	14.7%
Southern Asia	53	17.3%
South America	45	14.7%
South-Eastern Asia	43	14.0%
Eastern Africa	42	13.7%
Western Africa	38	12.4%
Central America	13	4.2%
Other	28	9.1%
**ESBL resistance mechanisms (n = 285)**	…	…
CTX-M-15	177	62.1%
CTX-M-55	32	11.2%
CTX-M-27	44	15.4%
CTX-M-14	16	5.6%
Other CTX-M	9	3.2%
Other ESBL	10	3.5%
**Colistin resistance mechanisms (n = 28)**	…	…
MCR-1	26	92.9%
MCR-3	3	10.7%
**CRE resistance mechanisms (n = 2)**	…	…
NDM-5	2	100.0%

### Acquired ESBL Resistance Mechanisms Vary by Geography

**Figure 3.**
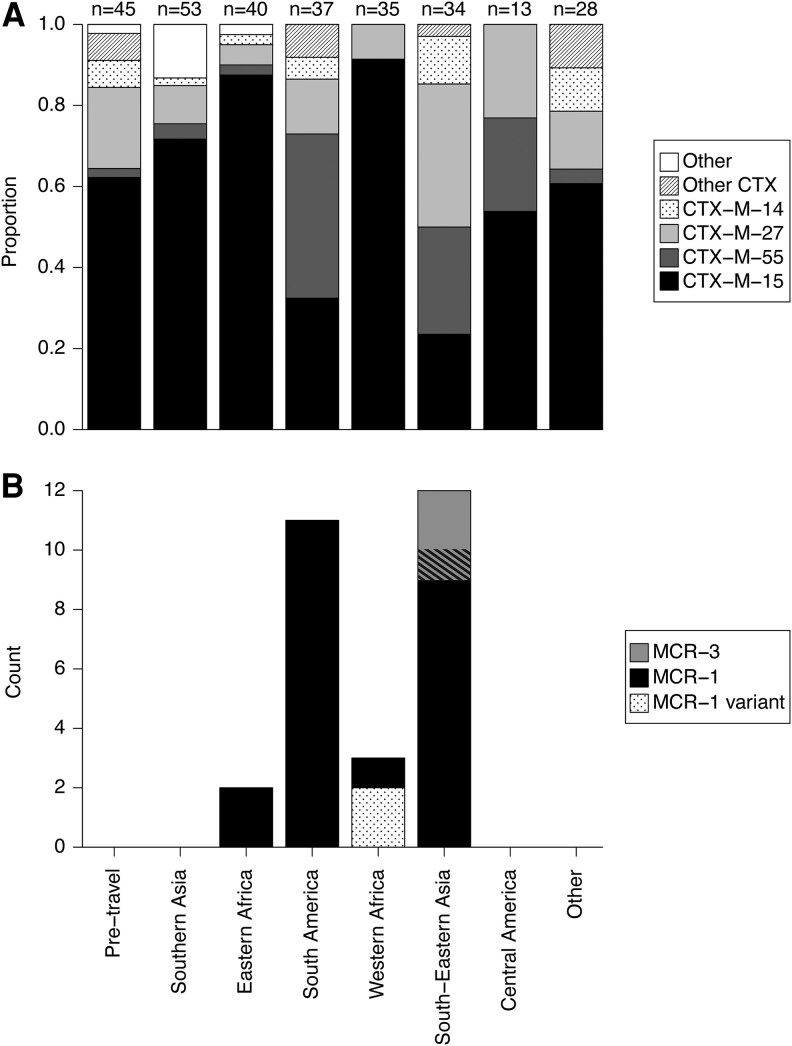
Resistance alleles show geographic heterogeneity. Genes mediating the observed resistance phenotypes are broken down by travel region. *A*, Relative proportion of ESBL genes by region. The 4 most common CTX-M alleles are shown. Total counts are given above bars; counts exceed the number of ESBL organisms due to multiple ESBL gene carriage in 3 isolates (Supplementary Table 1). *B*, *mcr* gene counts by region. One isolate carried both *mcr-1* and *mcr-3*. *mcr-1* variants differ from *mcr-1.1* by a single mutation.

Although we observed little phylogenetic clustering by travel region, suggesting a limited role of specific bacterial lineages in driving local AMR prevalences, we hypothesized that horizontal gene transfer in *E. coli* might allow ARGs to proliferate regionally on diverse genetic backgrounds. We therefore evaluated the resistance mechanisms associated with the targeted AMROs to identify any geographic associations. Most ESBL-PEs carried a *bla*_CTX-M_ gene, including 96% (43/45) of pre-travel and 97% (232/240) of post-travel ESBL-PE isolates (Figure 3*A*). The most common allele in both pre- and post-travel samples was *bla*_CTX-M-15_, though the allele distribution varied by geography. Compared with other regions, *bla*_CTX-M-27_ was significantly more common in isolates associated with South-Eastern Asia (OR = 3.80, 95% CI: 1.64, 8.83; FDR = 0.0092). *bla*_CTX-M-55_ was observed more frequently in isolates associated with South America (OR = 6.56, 95% CI: 2.61, 16.46, FDR = 0.00090) and South-Eastern Asia (OR = 3.71, 95% CI: 1.38, 9.99, FDR = 0.039) (Figure 3*B*). These findings are concordant with previous studies describing the expansion of *bla*_CTX-M-27_ in South-Eastern and Eastern Asia [29] and the global prevalence of *bla*_CTX-M-55_ [30]. There were 10 non-*bla*_CTX-M_ ESBL-PE isolates, 7 of which were collected from travelers returning from India. Resistance mechanisms in these isolates varied, including *bla*_CMY_ (n = 5) and *bla*_SHV_ (n = 3). Two unrelated *E. coli* isolates had no identified ESBL gene; resistance in these isolates was potentially mediated by *ampC* mutations. Two travelers acquired CRE during travel after visiting South-Eastern Asia (phylogroup C *E. coli*) and Southern Asia (phylogroup F *E. coli*); both isolates carried *bla*_NDM-5_ and *bla*_CTX-M-15_.

We next looked for associations between traveler behavior and ESBL gene carriage in post-travel isolates (*Methods*). In univariable models, isolates collected from travelers who reported eating raw or undercooked fish were significantly more likely to carry *bla*_CTX-M-55_ (OR = 6.99, 95% CI: 2.37, 20.62; FDR = 0.013), while those associated with drinking unpurified water were significantly more likely to carry *bla*_CTX-M-27_ (OR = 3.64, 95% CI: 1.61, 8.21; FDR = 0.031); however, neither result was significant after adjusting for travel destination (Supplementary Table 2).

In concordance with its known carriage on plasmids [30], *bla*_CTX-M-55_ was distributed broadly across the *E. coli* tree; at most, 3 *bla*_CTX-M-55_-carrying strains clustered within a common sequence type. In contrast, almost half (13/27; 48%) of the *bla*_CTX-M-27_-carrying strains belonged to ST131 (OR = 6.55 [95% CI: 2.93, 14.62]; *P* = 4.5×10^−6^), corresponding to clonal spread of a known epidemic subclade [31].

### Diverse Plasmids Drive ESBL Dissemination

Plasmids are important drivers of AMR dissemination, concordant with the minimal phylogenetic association observed between *E. coli* strains and ESBL alleles. To explore the role of plasmids and better characterize the genomic neighborhood of ESBL genes, we generated near complete hybrid assemblies for the 216 isolates with long-read sequence data available and classified plasmid content [32]. There was considerable diversity in the plasmid types associated with ESBL genes. For instance, *bla*_CTX-M-15_ was carried on at least 18 distinct plasmid groups. There was limited association between the ARG host plasmid group and travel region (Supplementary Figure 1*A*). Chromosomal carriage was more common for *bla*_CTX-M-14_ (43%) and *bla*_CTX-M-15_ (27%), and chromosomal integration of these genes was observed across all phylogroups (Supplementary Figure 1*B*).

Given the observed association between *bla*_CTX-M-55_ carriage and travel to South America and South-Eastern Asia, we sought to determine whether horizontal transfer of the same genomic sequence could be driving its circulation in these regions. Analysis of the *bla*_CTX-M-55_ genomic neighborhood revealed geographic patterns; the *bla*_CTX-M-55_ neighborhood in isolates associated with South-Eastern Asia was commonly ISEcp1-hp-*bla*_CTX-M-55_-hp-Tn2 (corresponding to ‘Type I’ in a previous study [30], n = 5/8) while isolates from travelers to South America typically followed the motif IS15-*bla*_CTX-M-55_-hp-(*bla*_TEM_)-IS26 (‘Type II’, n = 6/7) (Supplementary Figure 2). Previous analyses of *bla*_CTX-M-55_ contexts have shown type II to be increasing in prevalence, more common in genomes from South America, and often associated with animal sources [30].

### Colistin Resistance Gene Variants and Geographically Localized Plasmids Identified Among Traveler Samples

A total of 28 isolates were mcr-E; all were *E. coli* from post-travel samples, most often associated with travel to South America (n = 11) and South-Eastern Asia (n = 12). Three isolates carried *mcr-3*, all associated with travel to South-Eastern Asia, and 26 carried *mcr-1*, with one isolate carrying both (Figure 3*B*). Two isolates associated with travel to Western Africa (Liberia and Ghana) carried an *mcr-1.1* gene with point mutations in the start codon, with retained functionality likely due to a subsequent second start codon. While start codon mutations have previously been observed in *mcr-1* [33], the specific mutations observed here appeared to be rare. One variant (M1R; Liberia) had 13 perfect matches in the NCBI nt database (as of September 2023), 2 collected prior to 2020, while the other (M1I; Ghana) had no matches.

Two travelers independently visiting Peru returned with colistin-resistant *E. coli* strains, harboring highly similar IncI2 plasmids carrying *mcr-1*. The 60 and 61 kb plasmids shared 59 kb at 99.8% nucleotide identity with 2 indels. We found 3 other plasmids in public databases with query coverage and nucleotide identity at least as high as our identified pair. All 3 also originated from South America (Peru, Bolivia and Ecuador), had various bacterial hosts (*K. pneumoniae*, *Citrobacter braakii*, and *E. coli*), and were isolated from human urine, food, and chicken, respectively.

### Regional Variability in Additional ARGs

While we selected for specific AMROs in our study design, we profiled ARG content across all ESBL isolates [34] to identify other regionally enriched resistance genes (*Methods*; Supplementary Figure 3, Supplementary Table 3). The genes *floR* (OR = 9.62, 95% CI: 3.51, 26.35; FDR = 0.0018) and *fosA3* (OR = 28.56, 95% CI: 9.13, 89.27; FDR = 2.7×10^−6^), conferring resistance to phenicol and fosfomycin, respectively, were elevated in ESBL-PE isolates from travelers returning from South America. Isolates from both South America (OR = 4.98, 95% CI: 1.78, 13.90; FDR = 0.047) and Central America (OR = 8.05, 95% CI: 2.25, 28.80; FDR = 0.037) had higher levels of the aminoglycoside resistance gene *APH(3′)-Ia*. However, based on AST profiles, we found no significant regional variability in phenotypic AMR (Supplementary Figure 4); the discordance due in part to the existence of multiple resistance mechanisms per phenotype.

### Virulence Factor Carriage Highlights Associations With Ecology and Traveler Behavior

As AMROs inhabit a range of environmental niches with differential selective pressures, we hypothesized that their gene content may be associated with their route of acquisition. To assess this, we looked for virulence and stress factors associated with travel regions and traveler activities. The salmochelin siderophore system encoded by the gene cluster *iroBCDEN* was enriched in isolates associated with travel to South America (OR = 7.20 [95% CI: 2.45, 21.1], FDR = 0.0011), as was the colicin-V gene *cvaC* (OR = 11.42 [95% CI: 3.46, 37.71], FDR = 0.0044). The virulence genes *capU* (OR = 3.78 [95% CI: 1.95, 7.31], FDR = 0.0044) and *aaiC* (OR = 5.88 [95% CI: 2.33, 14.88], FDR = 0.0078) were both enriched in isolates associated with travel to Southern Asia. We did not observe any associations between travel destination and *E. coli* pathotypes, including enterohaemorrhagic and enteropathogenic *E. coli* (EHEC and EPEC), based on virulence marker genes (*Methods*). While our previous study noted that acquisition of AMROs was associated with self-reported diarrhea during travel [24], the rate of diarrhea was not elevated among travelers acquiring any specific pathotype. Among stress factors, metal resistance genes (the *sil*, and *ter* gene clusters, encoding resistance to silver and tellurite, respectively) were enriched in isolates linked to South-Eastern Asia (sil: OR = 3.98 [95% CI: 1.69, 9.33], FDR = 0.030; ter: OR = 4.17 [95% CI: 1.64, 10.61], FDR = 0.041) (Supplementary Figure 5, Supplementary Tables 4 and 5).

The salmochelin operon *iroBCDEN*, as well as the *cvaC* gene, enriched in isolates associated with South America, are among the markers for the ColV plasmid, a driver of avian pathogenic *E. coli* [35]. We used a previously described marker-based approach to define ColV plasmids in our isolate collection (Supplementary Text) and sought to determine whether these isolates may be linked to animal exposure in our data. A total of 15 ColV isolates were identified; 85% of these (12/14 with associated questionnaire responses) were collected from travelers reporting animal contact (OR = 7.45 [95% CI: 1.51, 36.69]; *P* = .014).

**Figure 4.**
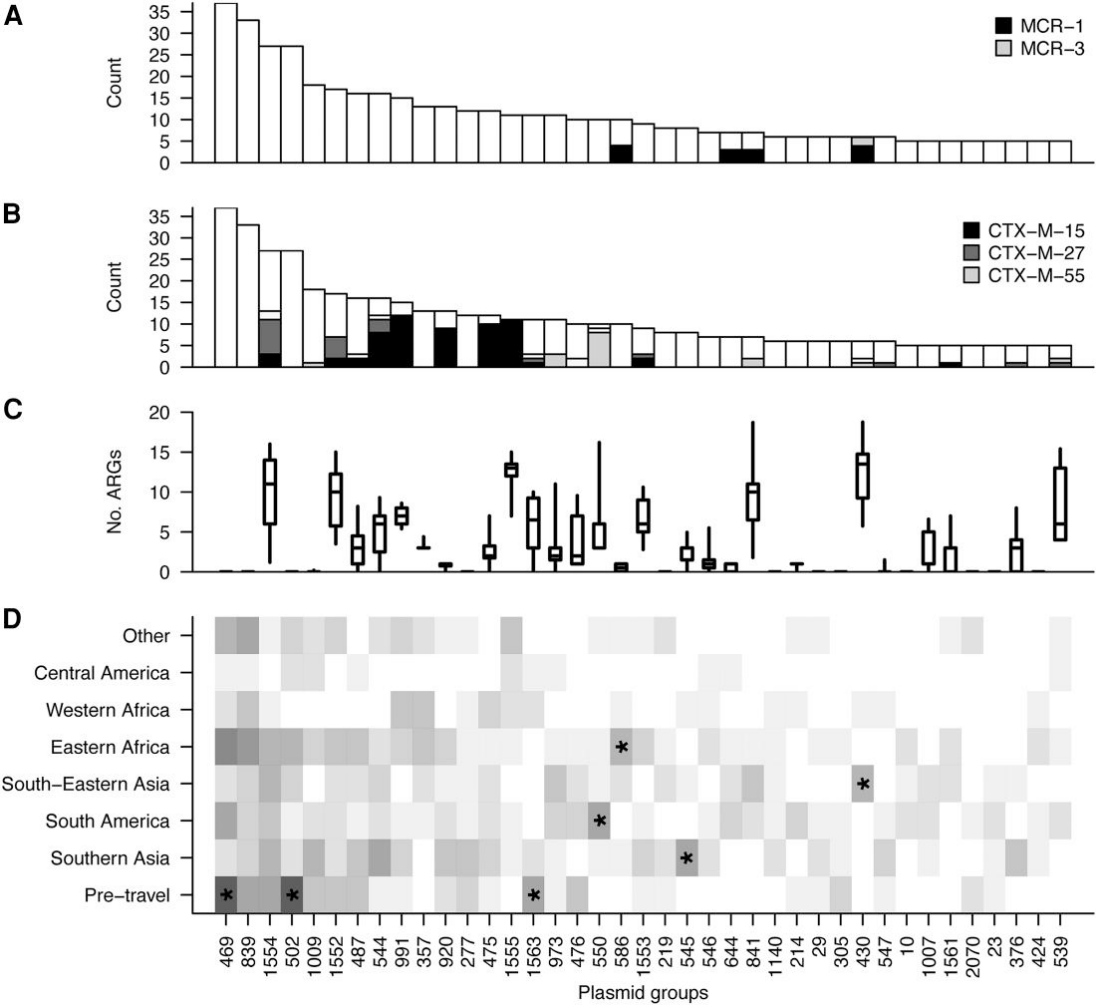
Plasmids vary in geographic distribution and ARG content. Plasmid content was identified with MOB-Suite for the 207 genomes with hybrid assemblies. *A*, Frequency of the most common plasmid clusters across all isolates, with MCR gene content marked. *B*, Frequency of the most common plasmid clusters across all isolates, with ESBL gene content marked. *C*, ARG count distribution for each of the plasmid groups. Box plots display the interquartile range and median, as well as the 95% quantiles. *D*, Heat map denoting frequency of plasmid group detection by travel region. Asterisks denote statistical significance after multiple testing correction; *FDR < 0.05 (see Supplementary Table 6).

Finally, we sought to determine if any potentially high-risk plasmids, such as ColV, were enriched in specific geographic regions, using the subset of hybrid-assembled isolates. While most plasmid groups were found across several travel destinations, some exhibited geographical enrichment, including plasmids belonging to group 430, which were significantly more common in isolates linked to South-Eastern Asia. These plasmids carried a median of 14 ARGs, including either *mcr-1* or *mcr-3* (Figure 4, Supplementary Table 6). Plasmid colocation of multiple ARGs represents elevated risk of rapid dissemination of multidrug resistance; detection of such plasmids highlights the power of traveler surveillance in combination with long read sequencing.

## DISCUSSION

Robust surveillance and analysis of circulating AMROs across global regions is essential for mitigating the growing threat of AMR. While considerable efforts are underway to establish global AMR surveillance networks [36], gaps remain. Implementing an international framework to collect appropriate samples is costly, resource intensive, and presents numerous logistical challenges. In contrast, surveillance of returning international travelers requires only local sample collection and processing, yet, provides a proxy insight into global AMR dynamics, complementing existing international surveillance efforts. We analyzed travel-acquired AMROs to explore global diversity and to identify regional patterns of resistance and virulence gene circulation, including potential emerging threats. The majority of acquired AMROs were highly diverse *E. coli* with limited phylogeographic signal, though many ARGs and virulence genes exhibited regional enrichment, driven by local plasmid-mediated dissemination.

To our knowledge, this is the largest study to date utilizing whole genome sequencing of travel-acquired AMROs to describe AMR patterns associated with travel regions; previous sequencing studies did not attempt to identify geographic patterns due to sample size or sampling approach [13, 16, 17, 20–24]. Several patterns identified in our study are concordant with previous analyses of global sample collections of *E. coli* from humans and wastewater, highlighting the utility of our indirect sampling via travelers. *E. coli* is known to be highly globally diverse, with limited geographic or environmental structure [37]. While we found *E. coli* ST131 more frequently in pre-travel samples, travel-associated strains exhibited considerable diversity regardless of destination. *bla*_CTX-M_ alleles vary by region, and their distribution has changed over time [29], with new variants emerging [38]. *bla*_CTX-M-55_ emerged in South-Eastern Asia [39], and was primarily found in Asia prior to 2017 [29, 39]. We also found several examples in travelers returning from South America, indicative of the subsequent global dissemination of this gene observed as of 2022, particularly to the Americas [30]. Characterizing the genomic neighborhood of this gene revealed that isolates from South America more frequently carried *bla*_CTX-M-55_ in a ‘type II’ background, which was more common in this region, and also linked to animal populations and food sources, based on a previous study of over 2000 *bla*_CTX-M-55_ isolates [30]. Travel-acquired CRE was rare, reflecting its strong association with healthcare exposure [40], and concordant with community-level studies which identified few CRE compared to ESBL-PE [41, 42].

We additionally found silver and tellurite resistance genes were more common in isolates associated with travel to South-Eastern Asia, potentially indicating increased exposure to industrial contamination, although further work is needed to explore these correlations. Finally, we identified a potential link between animal contact and elevated levels of ColV plasmid carriage in acquired AMROs, mirroring its known high prevalence among animal sources [43]. This highlights the potential for future traveler surveillance efforts to link acquired genes and their associated primary reservoirs.

Hybrid sequencing further allowed us to associate key genes with their diverse plasmid backgrounds. The ability to characterize the genomic context around target genes allows one to track mobile sequences across strains and plasmids and identify associations with particular environmental niches. We identified frequent ARG co-location on plasmids, including an *mcr*-carrying plasmid group associated with South-Eastern Asia harboring an average of 14 additional ARGs. Such plasmids pose a significant public health concern, and their dissemination should be monitored.

Travelers acquire AMROs at vastly different rates depending on destination; however, the utility of travelers as sentinels to report on regional genomic landscapes has yet to be explored. Such a traveler-based approach could identify potential threats emerging in regions with limited surveillance. This is highlighted in our study with the detection of rare mutations in the *mcr-1* gene in 2 travelers returning from Western Africa. Tracking phylogeographic patterns of clinically important genes is essential both for monitoring dissemination pathways, and the ability to rapidly respond to emerging threats.

Our cohort comprised only US travelers and may not be representative of destinations and activities of travelers more generally. While larger than previous studies of traveler AMROs, the sample size per travel region was relatively limited, hampering the ability to estimate prevalence of genomic features with precision, or to disentangle the many potential factors which might contribute to observed genome characteristics, contributing to non-significant results in multivariable models. Moreover, the aggregation of travel destinations into global regions may obscure important heterogeneity between and even within specific countries. Nevertheless, we were able to detect significant differences in resistance alleles and plasmids between regions, which could be explored at a more granular level in future studies. By picking morphologically distinct colonies from selective media, we captured greater diversity than a standard approach of taking a single isolate per individual, though we may still be missing additional, distinct strains. Since all studied isolates belonged to 3 specific AMR phenotypes, conclusions related to phylogenetic distributions, gene content, and plasmids are not necessarily generalizable, due to colocation or interaction with targeted ARGs. Finally, since travelers were recruited between 2017 and 2019, observed trends may have shifted following the COVID-19 pandemic, alongside changing patterns of antibiotic use globally [44].

While there have been efforts to expand global surveillance of AMR, including through multinational networks [45] and targeted community-level efforts [46–49], coverage remains sparse and requires extensive coordination to collect reliable samples. Given the nature of international travel, using travelers as sentinels could be an important approach to provide complementary AMR surveillance, including offering greater visibility in regions where AMR surveillance is limited. Such a framework would be ideally implemented prospectively to understand AMR trends in real time. Traveler pathogen genomic surveillance, if implemented systematically with geographic representation, could provide insight on global AMR dynamics, and importantly, provide information about global dissemination of emerging AMR threats.

## Supplementary Material

jiaf469_Supplementary_Data

## Supplementary Data

Supplementary materials are available at *The Journal of Infectious Diseases* online (http://jid.oxfordjournals.org/). Supplementary materials consist of data provided by the author that are published to benefit the reader. The posted materials are not copyedited. The contents of all supplementary data are the sole responsibility of the authors. Questions or messages regarding errors should be addressed to the author.
