# Supplementary material for: Insights Into Global Antimicrobial Resistance Dynamics Through the Sequencing of Enteric Bacteria From US International Travelers
Source: J Infect Dis. 2025 Sep 24;233(1):e164–73. doi: 10.1093/infdis/jiaf469 (PMC12811885; doi:10.1093/infdis/jiaf469)
Supplement: jiaf469_Supplementary_Data [file jiaf469_supplementary_data.zip › S4_ast_profiles_by_region.pdf]

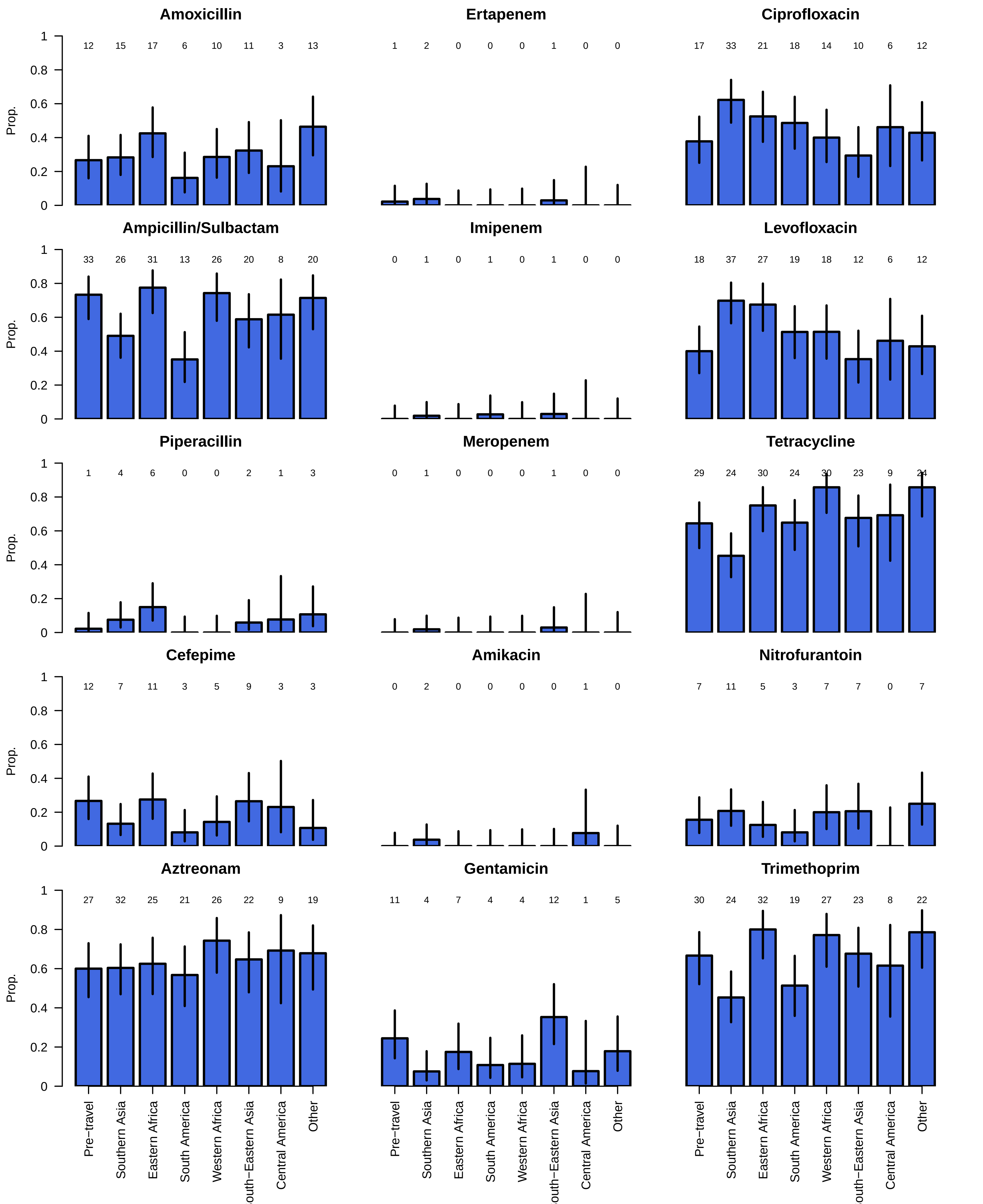

Amoxicillin
12 15 17 6 10 11 3 13
Ertapenem
1 2 0 0 0 1 0 0
Ciprofloxacin
17 33 21 18 14 10 6 12
Ampicillin/Sulbactam
33 26 31 13 26 20 8 20
Imipenem
0 1 0 1 0 1 0 0
Levofloxacin
18 37 27 19 18 12 6 12
Piperacillin
1 4 6 0 0 2 1 3
Meropenem
0 1 0 0 0 1 0 0
Tetracycline
29 24 30 24 30 23 9 24
Cefepime
12 7 11 3 5 9 3 3
Amikacin
0 2 0 0 0 0 1 0
Nitrofurantoin
7 11 5 3 7 7 0 7
Aztreonam
27 32 25 21 26 22 9 19
Gentamicin
11 4 7 4 4 12 1 5
Trimethoprim
30 24 32 19 27 23 8 22
Prop.
0 0.2 0.4 0.6 0.8 1
Pre−travel
Southern Asia
Eastern Africa
South America
Western Africa
outh−Eastern Asia
Central America
Other
