# Supplementary material for: Insights Into Global Antimicrobial Resistance Dynamics Through the Sequencing of Enteric Bacteria From US International Travelers
Source: J Infect Dis. 2025 Sep 24;233(1):e164–73. doi: 10.1093/infdis/jiaf469 (PMC12811885; doi:10.1093/infdis/jiaf469)
Supplement: jiaf469_Supplementary_Data [file jiaf469_supplementary_data.zip › SupplementaryInformation.docx]

## **Supplementary Information for “*Insights Into Global Antimicrobial Resistance Dynamics Through the Sequencing of Enteric Bacteria From US International Travelers*”**

Sridhar et al.

## **Supplementary Text: Full Methods**

### *Study design & sample collection*

We recruited participants at five U.S. travel clinics affiliated with Global TravEpiNet [1], located in Boston MA, New York NY, Bronx NY, Salt Lake City UT and LeHigh Valley, PA, during a pre-travel health visit, as previously described [2]. Participants were aged 1-85 years, and no exclusion criteria were applied. Recruitment began in 2017 at the Boston site, and in 2018 for all other sites, and concluded in 2020. Written informed consent was obtained from all participants in the study. Institutional review board approval was obtained from the human research committee at each participating enrollment site. Participants self-collected pre- and post-travel stool samples, which were immediately stored in Cary Blair medium and mailed to the Massachusetts General Hospital clinical microbiology laboratory. Benchmarking was performed to assess the recovery of AMR organisms under this sample collection protocol [3]. Samples were screened for the presence of extended spectrum beta-lactamase producing Enterobacterales (ESBL-PE), *mcr*-mediated colistin-resistant Enterobacterales (mcr-E) and carbapenem-resistant Enterobacterales (CRE) as previously described [3,4]. Briefly, cultures were screened for ESBL-PE by growth on HardyCHROM ESBL (Hardy Diagnostics, Santa Maria, CA) medium at 35°C and for CRE by either growth on McConkey agar following incubation in tryptic soy broth inoculated with a 10-µg meropenem disc [5] or growth on CHROMID CARBA (bioMerieux, Durham, NC) medium at 35°C. Colistin resistance was screened for using Luria Bertani agar containing colistin sulfate, vancomycin, and amphotericin [4]. Resistant colonies were picked from the selective screening; multiple colonies were picked if there were distinct morphotypes. Recovered organisms underwent initial antimicrobial susceptibility testing (AST) against a panel of 18 antibiotics using the Vitek2 antimicrobial susceptibility testing automated system (bioMérieux, Durham, NC); ampicillin, amoxicillin, ampicillin/sulbactam, piperacillin, cefazolin, ceftriaxone, cefepime, aztreonam, ertapenem, imipenem, meropenem, amikacin, gentamicin, ciprofloxacin, levofloxacin, tetracycline, nitrofurantoin, trimethoprim-sulfamethoxazole. Colistin AST was also performed using an internally validated broth microdilution panel (Sensititre, ThermoFisher Scientific, Waltham, MA). Ceftriaxone, carbapenem, and colistin non-susceptibility was confirmed using phenotypic (ESBL-PE, CRE) or molecular (mcr-E) testing as previously described [4]. Healthcare providers used structured questionnaires before travel to collect information on demographics, health, travel itineraries and activities, medications and symptoms. Travelers completed a questionnaire after travel regarding behaviors and illness while traveling.

### *Whole genome sequencing*

*DNA extraction.* 1 ml of a 4 ml overnight culture of each isolate grown in Luria Bertani broth was centrifuged for 10 minutes at 7500 rpm to pellet cells. The supernatant was discarded and pellets were processed using the Qiagen QIAamp DNA Mini kit. The only deviations from the standard protocol were that samples were not incubated at 70°C following addition of Buffer AL, and samples were eluted in 100 µl distilled water. DNA concentrations (ng/µl) were measured using a Nanodrop.

*Illumina sequencing.* Illumina whole genome paired-end libraries were prepared for a total of 393 isolates as previously described, and sequenced on Illumina HiSeq 2500 or HiSeq X sequencers at the Broad Institute [6].

*Oxford Nanopore sequencing.* 600 ng of DNA from each sample was used as input into the Oxford Nanopore 1D ligation library construction protocol (SQK-LSK109) following the manufacturer’s recommendation. Samples were barcoded using the Native Barcoding Expansion 1-12 kit to run in batches of between 1 and 10 samples per flow cell on a GridIon (Oxford Nanopore Technologies Ltd, Science Park, UK). Samples were run on flowcells FLO-MIN106D (R9.4.1) and base-called using ont-guppy-for-minknow v2.0.5. Due to resource limitations, ONT data were generated for 263 isolates, approximately representing the first two-thirds of isolates received. A median of 0.9Gb were generated per isolate, resulting in a median genome coverage of 188x. Reads reached a median N50 of 7.9kb.

### *Sequence data analysis*

*Assembly and annotation.* All Illumina short read datasets were assembled using Spades v3.11.1 [7], and annotated with Prokka v1.14.6 [8]. For the subset of isolates with long read data available, genomes were assembled using UniCycler v0.4.4 [9] pipeline described previously [10], and annotated using the Broad Institute’s prokaryotic annotation pipeline, as previously described [11].

*Removal of duplicate isolates.* While colonies were selected based visually on differential morphology, some sequenced isolates from the same sample were nearly identical. To avoid overrepresentation of strains with a recent common ancestor which may represent the same exposure event, we used read-based alignment against the GenBank reference genome CP015159.1 to calculate pairwise SNP distances between all isolates. Strain pairs from the same individual separated by <50 SNPs were considered to belong to the same lineage, and one isolate was removed from further consideration. If ONT data were available for one isolate, this was retained; if isolates represented both pre- and post-travel, the pre-travel isolate was retained; otherwise, one isolate was retained at random. A total of 86 isolates were removed on the basis of this filtering, leaving 307 isolates for analysis. Post filtering, seven pairs of isolates collected from the same individual belonged to the same sequence type; the pairwise distances ranged from 500-12,000 SNPs, and most exhibited distinct antimicrobial susceptibility profiles. These isolates were therefore considered distinct and were retained for analysis.

*Gene identification and pathotyping.* Resistance genes were identified using CARD and RGI v5.5.1 [12]. Virulence and stress factors were identified using AMRFinderPlus v3.11.2 [13]. Many entries are part of operons, and are thus highly correlated in presence. We identified virulence and stress factors with pairwise correlation coefficients >0.8 and collapsed these into clusters for downstream analysis; in almost all cases these clusters were concordant with known operons. An isolate was assumed to have an operon present if more than half of its constituent genes were identified.

Pathotypes were determined based on presence of pathotype-associated virulence genes; these were determined to be those most commonly used for pathotype classification in the literature [14–16]. Enteropathogenic *E. coli* (EPEC) were defined as isolates containing genes *eae* or *bfp*; enteroaggregative *E. coli* (EAEC) were those containing *agg*, *aat*, or *aai*; diffusely adherent *E. coli* (DAEC) were those containing *afa* or *dra*; enteroinvasive *E. coli* (EIEC) were those containing *ipa*; enterotoxigenic *E. coli* (ETEC) were those containing *elt* or *est*; and enterohaemorrhagic *E. coli* (EHEC) were those containing *stx*. Any isolates not containing any of these genes were defined as non-diarrheagenic (non-DEC) *E. coli*.

As previously defined [17], we considered an isolate ColV-positive if it carried at least one or more genes from four or more of the following six gene sets (i) *cvaABC* and *cvi* (the ColV operon), (ii) *iroBCDEN* (salmochelin), (iii) *iucABCD* and *iutA* (aerobactin), (iv) *etsABC*, (v) *ompT* and *hlyF*, and (vi) *sitABCD*. Since not all of these genes belong to the AMRFinderPlus database, we used BLAST to identify relevant hits to the Virulence Factor Database [18].

*Phylogenetic tree construction.* Core genome alignment was performed using Parsnp (Treangen et al. 2014) on all 292 *E. coli* isolate assemblies. Recombination removal and tree construction was performed using Gubbins [19] with RAxML [20]. The *E. coli* phylogeny was visualized in iTOL [21] using midpoint rooting.

*Plasmid classification.* Plasmid content within the hybrid assemblies was classified using MOB-Suite version v1.4.9 [22]. Plasmids were assessed for pairwise shared, contiguous content using ConSequences [10].

### *Statistical analysis*

Travel destination was provided at country level; to increase per-destination sample size, we aggregated travel destinations into geographic regions according to the United Nations Statistics Division M49 standard (subregion level) [23]. We grouped regions with fewer than 10 travelers together into an ‘Other’ category. We hypothesized that travel-acquired isolates are representative of the microbial communities circulating in the travel destination, and that gene content may be linked to specific environmental niches, for which self-reported traveler activities can be a proxy. All statistical tests were performed at the ‘isolate-level’ rather than the traveler-level, since we were primarily interested in the characteristics of the microbial populations associated with geographic regions and environmental niches. Although we filtered out highly similar isolates, analyzing multiple isolates from the same host may still result in biases due to non-independence. To account for multiple isolates from the same host, we used generalized estimating equations (GEE) to test for associations between binary features (e.g. gene presence, plasmid presence, antibiotic non-susceptibility) and both geographic region and traveler activities. Activity variables considered were: swimming in ocean, swimming in lake/river/stream, swimming in a pool, eating undercooked fish, eating undercooked red meat, eating unpeeled fruit, eating uncooked vegetables, eating street food, dining in the home of friends/family, drinking unpurified water, working, animal contact, and visiting a healthcare facility. We used the package ‘geepack’ [24] in R v4.2.1 to fit GEE models and calculate odds ratios, confidence intervals and p-values. Multiple isolates per traveler represent unordered repeated observations with no anticipated differences in correlation between samples; as such, we used an exchangeable correlation structure. When exploring associations between genomic features and geographic regions, all isolates were included in analyses, including ‘pre-travel’ as a category. Associations between genomic features and traveler activities were tested using the set of post-travel isolates only. Models were fit for all gene-region or gene-activity pairs, and genes were collapsed into operon clusters where appropriate (see *Gene identification and pathotyping*). All genes with prevalence between 5% and 75% were included. The Benjamini-Hochberg procedure was performed on each set of results to control for global false discovery; resistance, virulence and stress genes were considered separately.

##

## **References**

1. LaRocque RC, Rao SR, Lee J, Ansdell V, Yates JA, Schwartz BS, et al. Global TravEpiNet: a national consortium of clinics providing care to international travelers--analysis of demographic characteristics, travel destinations, and pretravel healthcare of high-risk US international travelers, 2009-2011. Clin Infect Dis. 2012;54: 455–462.

2. Worby CJ, Earl AM, Turbett SE, Becker M, Rao SR, Oliver E, et al. Acquisition and Long-term Carriage of Multidrug-Resistant Organisms in US International Travelers. Open Forum Infect Dis. 2020;7: ofaa543.

3. Turbett SE, Becker M, Desrosiers L, Oliver E, Rao SR, Walker AT, et al. The effect of transport temperature and time on the recovery of antimicrobial-resistant Enterobacterales in stool. Diagn Microbiol Infect Dis. 2021;99: 115210.

4. Turbett SE, Desrosiers L, Andrews-Dunleavey C, Becker M, Walker AT, Esposito D, et al. Evaluation of a Screening Method for the Detection of Colistin-Resistant Enterobacteriaceae in Stool. Open Forum Infect Dis. 2019;6: ofz211.

5. Laboratory Protocol for Detection of Carbapenem-Resistant or Carbapenemase-Producing, Klebsiella spp. and E. coli from Rectal Swabs. [cited 13 Jan 2025]. Available: https://www.aab.org/images/aab/pdf/2013/Klebsiella_or_Ecoli_Lab_protocol.pdf

6. Ribeiro FJ, Przybylski D, Yin S, Sharpe T, Gnerre S, Abouelleil A, et al. Finished bacterial genomes from shotgun sequence data. Genome Res. 2012;22: 2270–2277.

7. Bankevich A, Nurk S, Antipov D, Gurevich AA, Dvorkin M, Kulikov AS, et al. SPAdes: a new genome assembly algorithm and its applications to single-cell sequencing. J Comput Biol. 2012;19: 455–477.

8. Seemann T. Prokka: rapid prokaryotic genome annotation. Bioinformatics. 2014;30: 2068–2069.

9. Wick RR, Judd LM, Gorrie CL, Holt KE. Unicycler: Resolving bacterial genome assemblies from short and long sequencing reads. PLoS Comput Biol. 2017;13: e1005595.

10. Salamzade R, Manson AL, Walker BJ, Brennan-Krohn T, Worby CJ, Ma P, et al. Inter-species geographic signatures for tracing horizontal gene transfer and long-term persistence of carbapenem resistance. Genome Med. 2022;14: 37.

11. Cerqueira GC, Earl AM, Ernst CM, Grad YH, Dekker JP, Feldgarden M, et al. Multi-institute analysis of carbapenem resistance reveals remarkable diversity, unexplained mechanisms, and limited clonal outbreaks. Proc Natl Acad Sci U S A. 2017;114: 1135–1140.

12. Alcock BP, Raphenya AR, Lau TTY, Tsang KK, Bouchard M, Edalatmand A, et al. CARD 2020: antibiotic resistome surveillance with the comprehensive antibiotic resistance database. Nucleic Acids Res. 2020;48: D517–D525.

13. Feldgarden M, Brover V, Gonzalez-Escalona N, Frye JG, Haendiges J, Haft DH, et al. AMRFinderPlus and the Reference Gene Catalog facilitate examination of the genomic links among antimicrobial resistance, stress response, and virulence. Sci Rep. 2021;11: 12728.

14. Boisen N, Østerlund MT, Joensen KG, Santiago AE, Mandomando I, Cravioto A, et al. Redefining enteroaggregative Escherichia coli (EAEC): Genomic characterization of epidemiological EAEC strains. PLoS Negl Trop Dis. 2020;14: e0008613.

15. Pakbin B, Brück WM, Rossen JWA. Virulence Factors of Enteric Pathogenic Escherichia coli: A Review. Int J Mol Sci. 2021;22. doi:10.3390/ijms22189922

16. Denamur E, Clermont O, Bonacorsi S, Gordon D. The population genetics of pathogenic Escherichia coli. Nat Rev Microbiol. 2021;19: 37–54.

17. Liu CM, Stegger M, Aziz M, Johnson TJ, Waits K, Nordstrom L, et al. Escherichia coli ST131-22 as a Foodborne Uropathogen. mBio. 2018;9. doi:10.1128/mBio.00470-18

18. Liu B, Zheng D, Zhou S, Chen L, Yang J. VFDB 2022: a general classification scheme for bacterial virulence factors. Nucleic Acids Res. 2022;50: D912–D917.

19. Croucher NJ, Page AJ, Connor TR, Delaney AJ, Keane JA, Bentley SD, et al. Rapid phylogenetic analysis of large samples of recombinant bacterial whole genome sequences using Gubbins. Nucleic Acids Res. 2015;43: e15.

20. Stamatakis A, Aberer AJ, Goll C, Smith SA, Berger SA, Izquierdo-Carrasco F. RAxML-Light: a tool for computing terabyte phylogenies. Bioinformatics. 2012;28: 2064–2066.

21. Letunic I, Bork P. Interactive Tree Of Life (iTOL) v5: an online tool for phylogenetic tree display and annotation. Nucleic Acids Res. 2021;49: W293–W296.

22. Robertson J, Nash JHE. MOB-suite: software tools for clustering, reconstruction and typing of plasmids from draft assemblies. Microb Genom. 2018;4. doi:10.1099/mgen.0.000206

23. United Nations Statistics Division. Methodology. [cited 19 Jan 2025]. Available: https://unstats.un.org/unsd/methodology/m49/

24. Højsgaard S, Halekoh U, Yan J. The R Package geepack for Generalized Estimating Equations. J Stat Soft. 2006;15: 1–11.

## **Supplementary Figure Legends**

**Figure S1. Target resistance genes exhibit diverse genomic backgrounds.** For each of the most common resistance genes conferring the observed resistance phenotype, its genomic context (plasmid group or chromosome) is given for all hybrid-assembled isolates, broken down by (a) travel region and (b) *E. coli* phylogroup. Numeric identifiers on the y-axis refer to MOB-Suite plasmid groups, which are listed individually when observed at least three times. ‘Other’ denotes plasmid groups with fewer than three observations, or ‘novel’ plasmid clusters, as determined by MOB-Suite (total number of unique observed groups provided in parentheses), while ‘Chr’ denotes chromosomal carriage.

**Figure S2. Specific CTX-M-55 genomic context varies by travel region.** The plasmid genomic neighborhood for all occurrences of CTX-M-55 in hybrid-assembled isolates. Travel region is given on the y-axis. All genes annotated with Prokka within 10kb of CTX-M-55 are depicted; resistance genes are colored red, transposons and insertion sequences are blue, all other annotated genes are green.

**Figure S3. Geographic and phylogroup distribution of antibiotic resistance genes.** Geographic and phylogenetic associations with resistance genes among ESBL organisms. All resistance genes identified by RGI in between 5-75% of isolates are included; the observed frequency of each gene is given in the bottom panel. Relative enrichment and significance are given for each gene vs. region (top panel). Cells are shaded by the odds ratio of gene presence in isolates from a given region vs. all other regions; red denotes OR>1, blue denotes OR<1. Gene-region pairs with no observations are denoted with ‘x’. * FDR<0.05 (See **Table S3** for full results).

**Figure S4. Antibiotic susceptibility by region.** For 15 antibiotics, the proportion of non-susceptible isolates (classified as ‘resistant’ or ‘intermediate’ based on Vitek2 AST results) is provided for each travel region. Counts of non-susceptible isolates are included above the bars.

**Figure S5. Prevalence of virulence and stress factors by region.** Geographic associations with virulence factors (left) and stress factors (right). All virulence and stress factors identified by AMRFinderPlus in between 5-75% of isolates are included; the observed frequency of each gene is given in the bottom panel. Relative enrichment and significance are given for each gene vs. region (upper panels). Cells are shaded by the odds ratio of gene presence in isolates from a given region vs. all other regions; red denotes OR>1, blue denotes OR<1. Gene-region pairs with no observations are denoted with ‘x’. * FDR<0.05 (See **Tables S4-S5** for full results).
