# Supplementary figures and images for: Insights Into Global Antimicrobial Resistance Dynamics Through the Sequencing of Enteric Bacteria From US International Travelers
Source: J Infect Dis. 2025 Sep 24;233(1):e164–73. doi: 10.1093/infdis/jiaf469 (PMC12811885; doi:10.1093/infdis/jiaf469)

**A**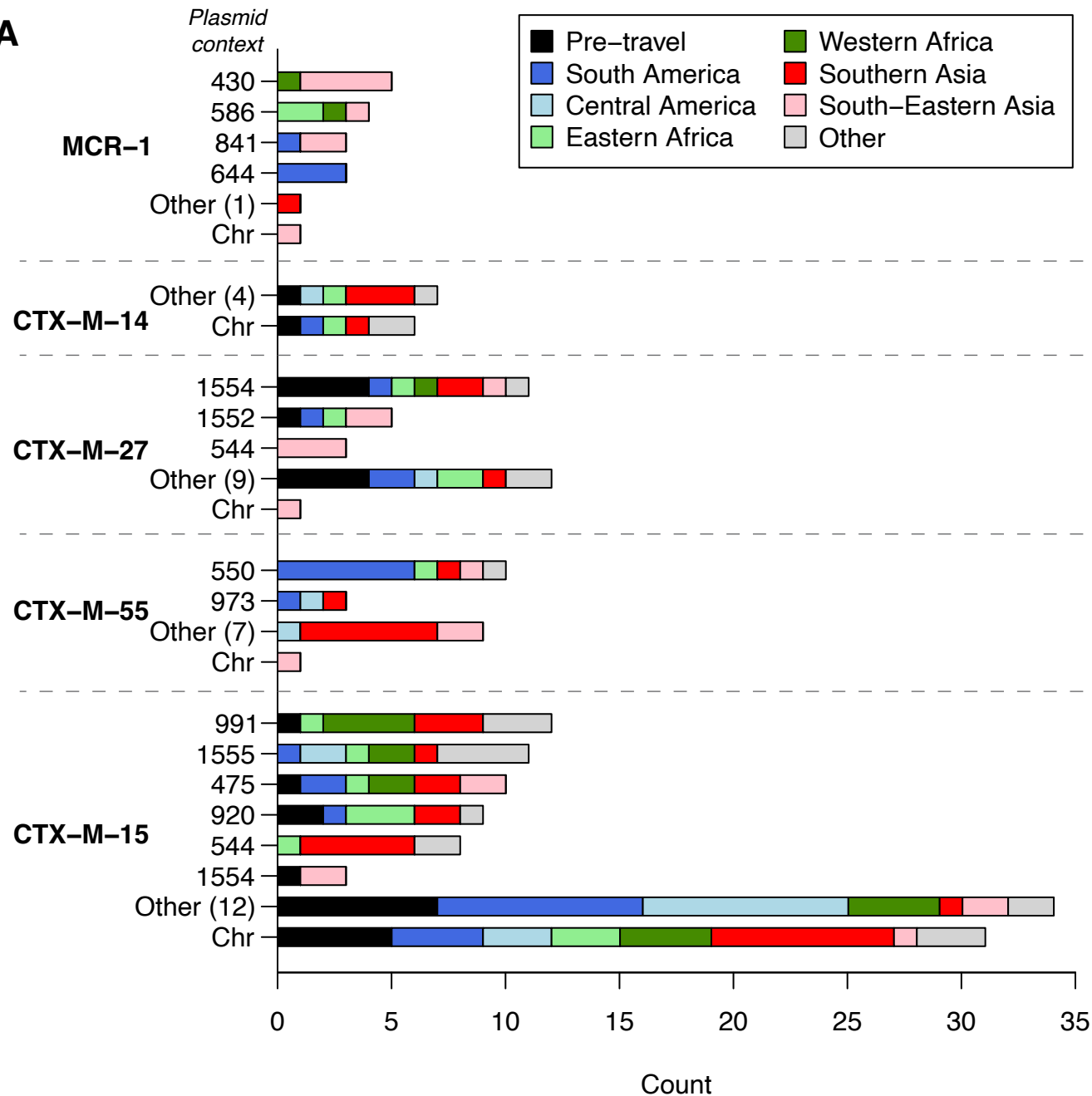**B**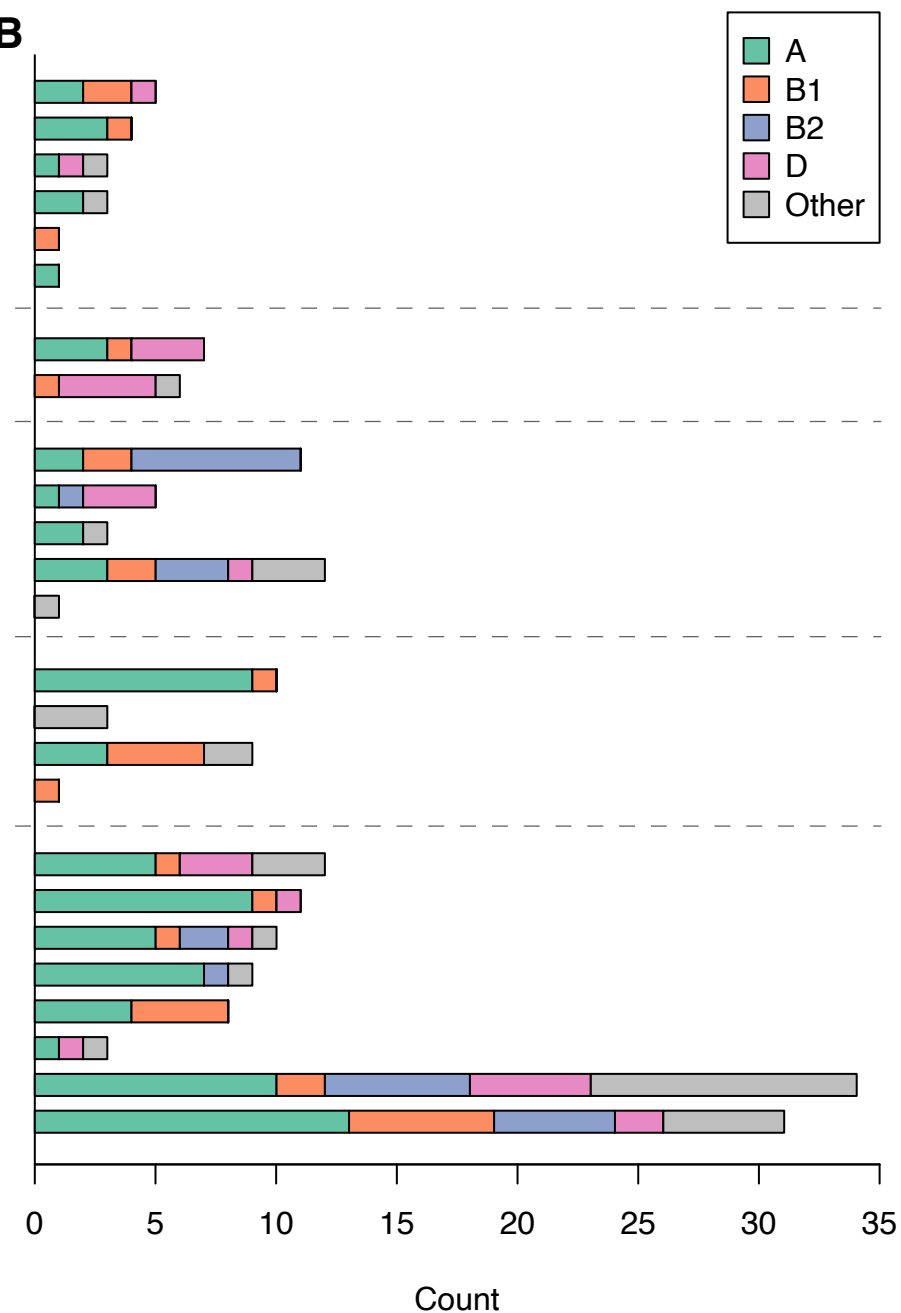

Supplement: jiaf469_Supplementary_Data [file jiaf469_supplementary_data.zip › S1_targetgene_plasmid.pdf]

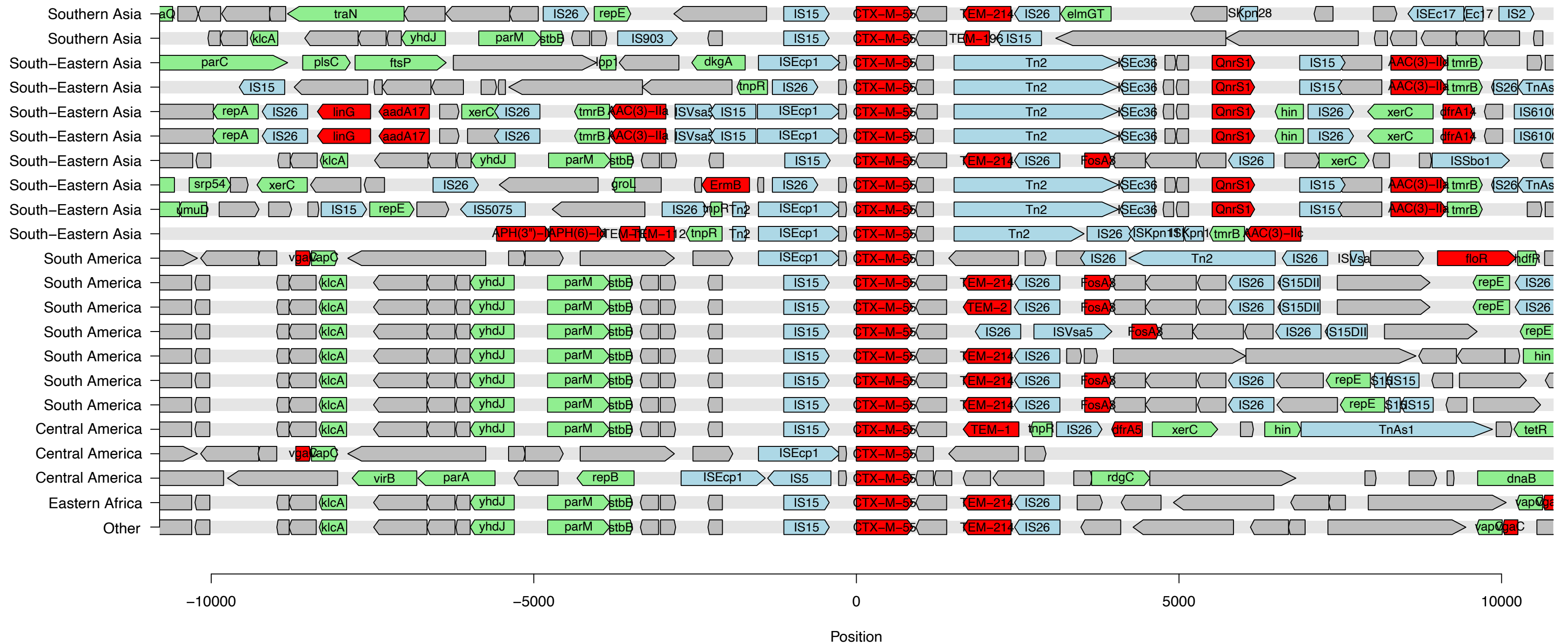

Supplement: jiaf469_Supplementary_Data [file jiaf469_supplementary_data.zip › S2_ctxm55contexts.pdf]

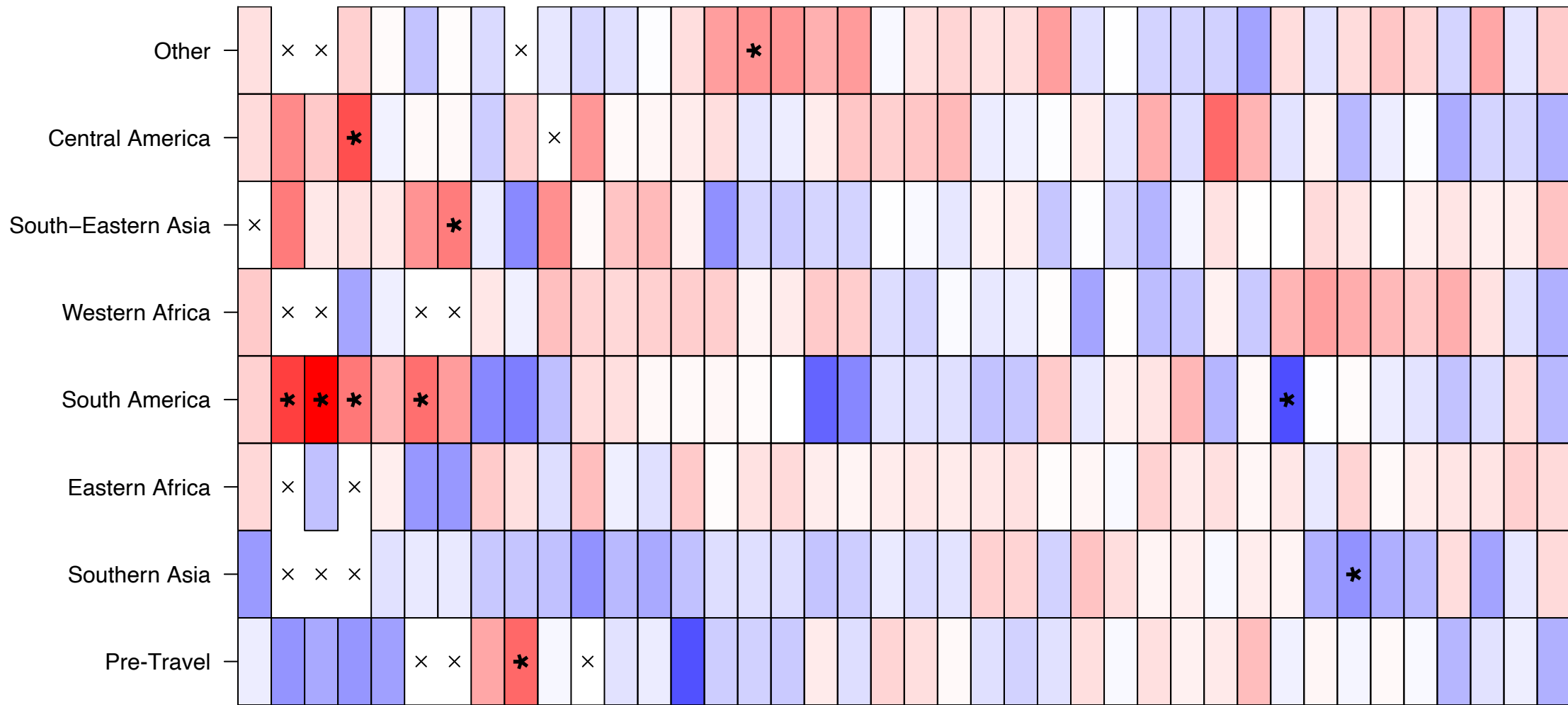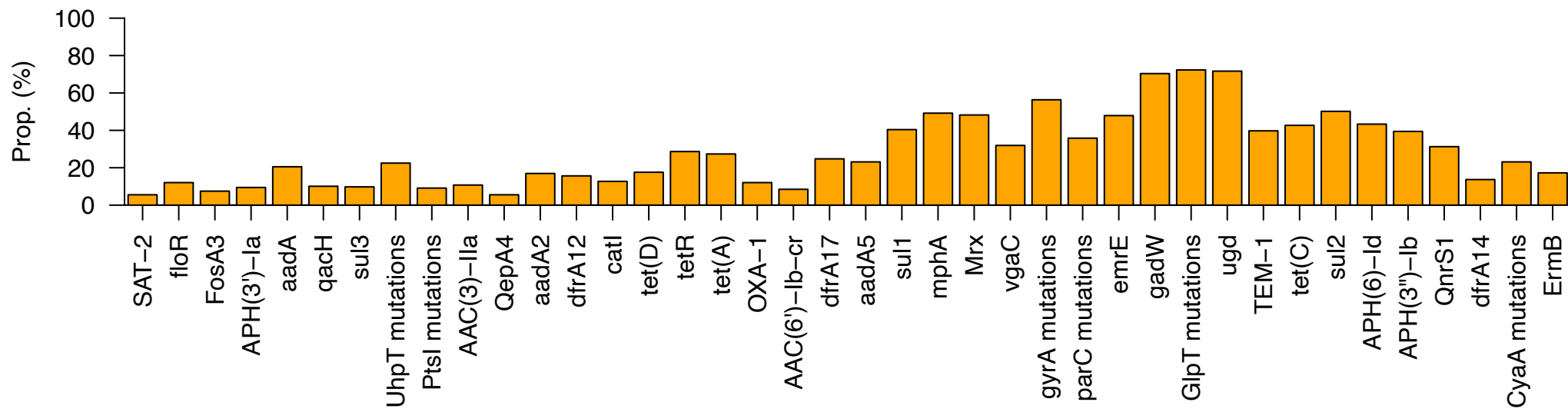

Supplement: jiaf469_Supplementary_Data [file jiaf469_supplementary_data.zip › S3_ARG_regions.pdf]

Virulence Factors

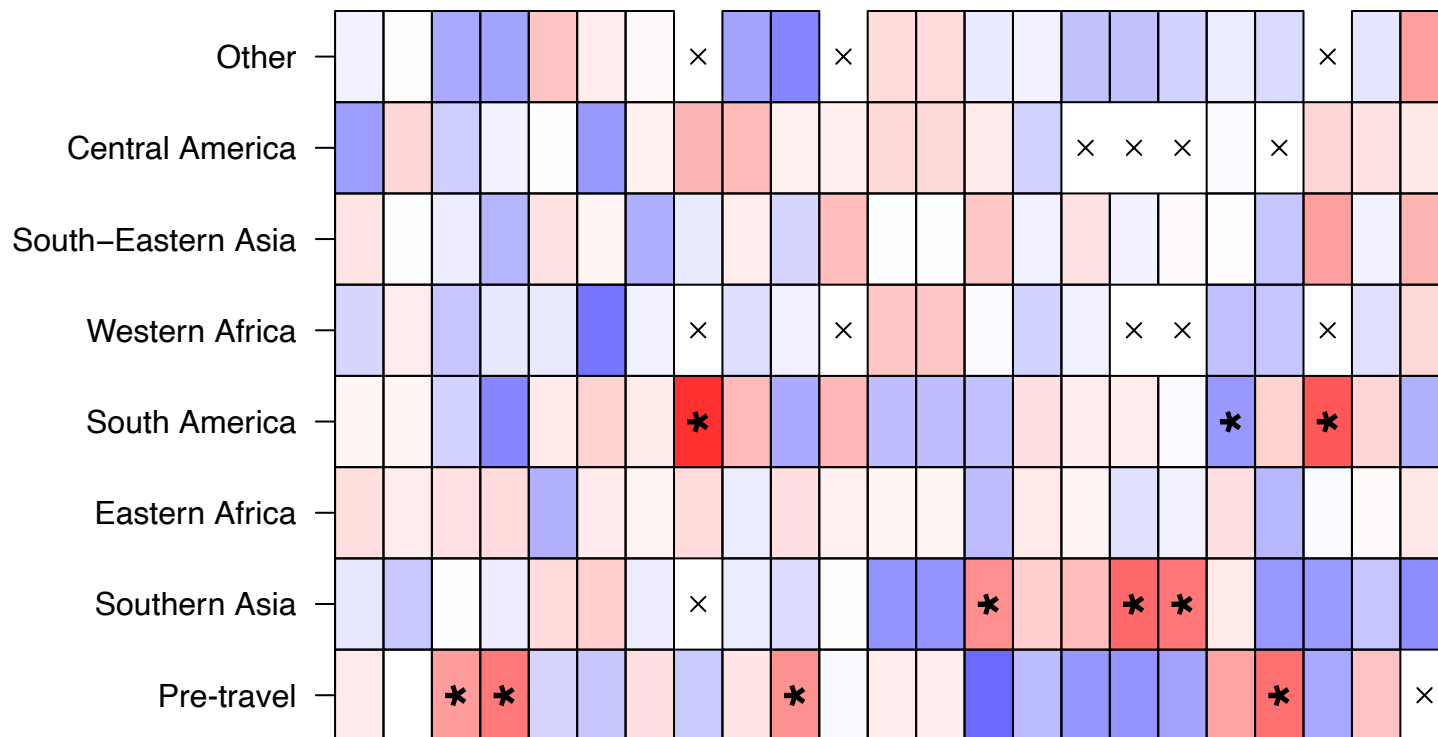

Stress Factors

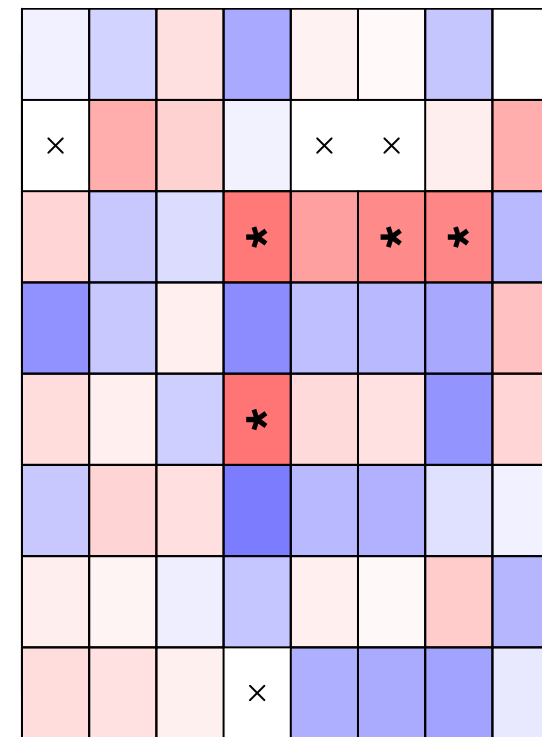

Prop. (%)

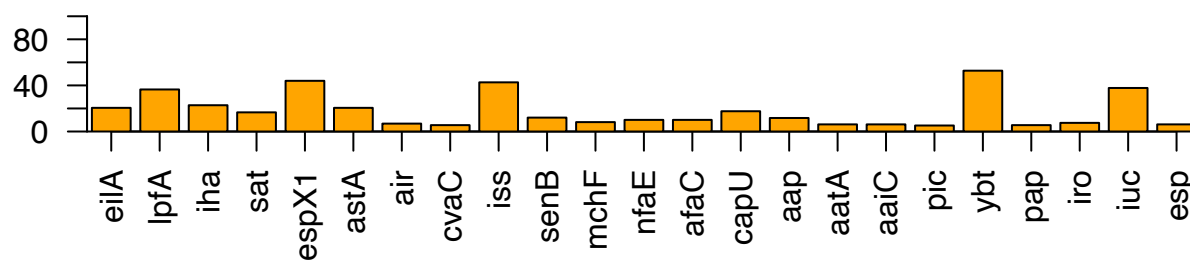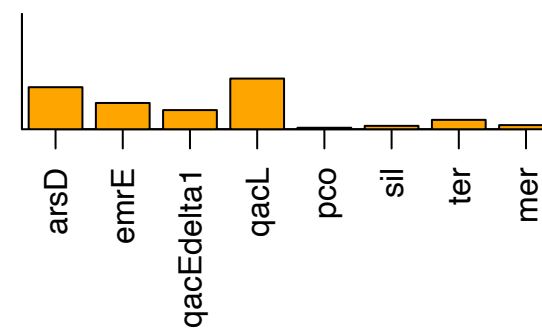

Supplement: jiaf469_Supplementary_Data [file jiaf469_supplementary_data.zip › S5_VFSF_regions.pdf]
